# Differential Expression of LMNA/C and Insulin Receptor Transcript Variants in Peripheral Blood Mononuclear Cells of Leukemia Patients

**DOI:** 10.3390/jcm13092568

**Published:** 2024-04-27

**Authors:** Khalid Saud Alshaalan, Turki Khalid Albawardi, Mahmoud Zhra, Norah Bin Sulaiman, Osama Yaheia Jnied, Rimah Abdullah Saleem, Ahmad Aljada

**Affiliations:** 1 College of Medicine, King Saud bin Abdulaziz University for Health Sciences, Riyadh 11481, Saudi Arabia; 2 Department of Biochemistry and Molecular Medicine, College of Medicine, Alfaisal University, Riyadh 11533, Saudi Arabia; mzahra@alfaisal.edu (M.Z.);

**Keywords:** mononuclear cells, leukemia, LMNA/C transcript variants, insulin receptor transcript variants

## Abstract

**Background**: Recent research has identified alternative transcript variants of LMNA/C (LMNA, LMNC, LMNAΔ10, and LMNAΔ50) and insulin receptors (INSRs) as potential biomarkers for various types of cancer. The objective of this study was to assess the expression of LMNA/C and INSR transcript variants in peripheral blood mononuclear cells (PBMCs) of leukemia patients to investigate their potential as diagnostic biomarkers. **Methods**: Quantitative *TaqMan* reverse transcriptase polymerase chain reaction (RT-qPCR) was utilized to quantify the mRNA levels of LMNA/C (LMNA, LMNC, LMNAΔ10, and LMNAΔ50) as well as INSR (IR-A and IR-B) variants in PBMCs obtained from healthy individuals (n = 32) and patients diagnosed with primary leukemias (acute myeloid leukemia (AML): n = 17; acute lymphoblastic leukemia (ALL): n = 8; chronic myeloid leukemia (CML): n = 5; and chronic lymphocytic leukemia (CLL): n = 15). **Results**: Only LMNA and LMNC transcripts were notably present in PBMCs. Both exhibited significantly decreased expression levels in leukemia patients compared to the healthy control group. Particularly, the LMNC:LMNA ratio was notably higher in AML patients. Interestingly, IR-B expression was not detectable in any of the PBMC samples, precluding the calculation of the IR-A:IR-B ratio as a diagnostic marker. Despite reduced expression across all types of leukemia, IR-A levels remained detectable, indicating its potential involvement in disease progression. **Conclusions**: This study highlights the distinct expression patterns of LMNA/C and INSR transcript variants in PBMCs of leukemia patients. The LMNC:LMNA ratio shows promise as a potential diagnostic indicator for AML, while further research is necessary to understand the role of IR-A in leukemia pathogenesis and its potential as a therapeutic target.

## 1. Introduction

Leukemia, a complex group of disorders consisting of myelogenous and lymphocytic types with acute and chronic subtypes, has undergone significant changes in its classification. These changes involve the inclusion of genetic and immunologic features in addition to morphology, resulting in a more comprehensive understanding of the disease and the role of genetic mutations [1,2,3]. Immunophenotyping, cytogenetics, and molecular analysis have become essential in the diagnosis and classification of leukemia [4,5], enhancing our ability to accurately categorize the disease for targeted treatments.

The use of monoclonal antibodies in immunologic classification has greatly advanced our knowledge of leukocyte differentiation and the origins of leukemia [6]. This approach has played a crucial role in identifying distinct phenotypes and subtypes [6]. For example, in adult acute lymphocytic leukemia, the presence of myeloid markers has been linked to poorer outcomes, highlighting the significance of precise classification based on specific markers [7]. Ongoing research is exploring the application of high-throughput sequencing to further improve the accuracy of classification [8], alongside advancements in diagnostic and therapeutic strategies that have significantly enhanced the prognosis of leukemia patients [8].

Cytogenetic analysis and molecular genetics have greatly advanced the classification and treatment of leukemias [9,10,11]. These methods are essential for identifying specific chromosomal abnormalities and genetic mutations, providing valuable prognostic information and guiding targeted therapies. They have played crucial roles in diagnosing, risk-stratifying, and monitoring therapy responses in pediatric and adult leukemias [10,11], leading to more effective treatment strategies based on the identification of specific genetic markers [9].

Nuclear lamins, classified as type V intermediate filaments, are crucial for nuclear stability and the regulation of nuclear processes such as DNA replication, transcription, and chromatin organization [12,13]. The *LMNA/C* gene, which encodes these proteins, has 12 exonal splicing variants, some of which are linked to cancer [14]. The loss of *LMNA/C* expression has been observed in breast cancer, lung cancer, leukemia, and lymphoma, while its up-regulation has been noted in prostate and colorectal cancer [15]. In solid tumors, the LMNC:LMNA mRNA ratio has been suggested as a potential biomarker [16,17]. However, the expression of LMNA/C transcript variants in leukemia remains unexplored.

Insulin signaling involves two main pathways. The first pathway operates through the phosphatidylinositol 3-kinase (PI3K)/AKT (protein kinase B (PKB)) pathway, responsible for producing insulin’s metabolic effects and connected through insulin receptor substrates (IRSs). The second pathway involves the Raf/Ras/MEK/MAPK (mitogen-activated protein kinase, also known as extracellular signal-regulated kinase (ERK)), pathway, controlling gene expression and, in conjunction with the PI3K pathway, regulating cell growth and differentiation [18]. The INSR plays significant roles in cancer progression, with overexpression in cancer cells leading to increased sensitivity to hyperinsulinemia [19]. The complex interplay between the INSR and tyrosine kinase receptors, such as growth factor receptors, and RAS pathway activation has significant implications for leukemia pathogenesis [20]. Dysregulated signaling through these pathways can lead to abnormal cell growth, survival, and differentiation, all characteristic features of leukemia development [21]. For instance, mutations or the overexpression of tyrosine kinase receptors like EGFR can directly activate the RAF/RAS/MEK/MAPK pathway, promoting the proliferation and survival of leukemic cells [22]. Additionally, the abnormal signaling of INSR can contribute to metabolic changes in leukemia cells, further propelling disease progression [23]. The significance of INSR in cancer is further emphasized by their overexpression in various human malignancies, particularly IR-A, and their involvement in the formation of hybrid receptors with the IGF-IR [24,25]. These hybrid receptors display enhanced ligand-binding affinity and signaling activity, leading to increased cancer cell proliferation and survival. Furthermore, the presence of these hybrid receptors confers resistance to targeted therapies that specifically target either INSR or the IGF-IR alone [24,26]. A thorough understanding of these signaling networks and their dysregulation in leukemia can offer valuable insights into potential therapeutic targets aimed at disrupting oncogenic signaling and improving treatment outcomes for leukemia patients [27].

The INSR gene undergoes alternative splicing, leading to the production of two mature isoforms with distinct structural characteristics. IR-A, which lacks exon 11, is widely expressed and is the predominant isoform in lymphocytes, while IR-B retains exon 11 and is responsible for mediating the metabolic effects of insulin in insulin-sensitive tissues like muscle, liver, and adipose tissues [28]. IR-A and IR-B play different roles in cancer. IR-A is often overexpressed in cancer and is linked to an increased risk of malignancies, whereas IR-B is typically found in insulin-sensitive tissues. The upregulation of IR-A enhances the mitogenic response of cancer cells to insulin and IGF-2, indicating that these isoforms could serve as potential targets for anti-cancer therapies [25,29,30]. Additionally, IR-A and IR-B exhibit differences in their ligand-binding capabilities and signaling pathways, with IR-A being more mitogenic and antiapoptotic [31]. The tissue-specific expression of these isoforms is regulated at both the mRNA and protein levels [32]. The ratio of IR-A:IR-B has been investigated in solid tumors like breast adenocarcinoma [30,33] and non-small cell lung cancers (NSCLC) [34] and has been proposed as a potential marker for predicting cancer progression and response to treatment.

PCR testing of biomarkers, such as transcript variants of genes like LMNA/C and INSR, presents numerous challenges and limitations. These include the need for meticulous primer design and validation, the difficulty of obtaining high-quality RNA samples, and the potential for false-negative and false-positive results [35,36]. The reproducibility and reliability of PCR assays across different laboratories and platforms also require rigorous validation and standardization protocols [37]. Furthermore, the clinical relevance and utility of these biomarkers often require additional validation through correlation studies with clinical outcomes and patient characteristics [38]. Despite these challenges, the potential of PCR in detecting transcript variants is significant, and further improvements in PCR techniques and protocols are necessary to enhance sensitivity and specificity [39].

In this study, we utilized *TaqMan* RT-qPCR to evaluate the mRNA expression levels of various transcript variants of LMNA/C (LMNA, LMNC, LMNAΔ10, and LMNAΔ50) and INSR (IR-A and IR-B) in PBMCs collected from healthy subjects and individuals with the four primary types of leukemia (AML, ALL, CML, and CLL). The objective was to investigate the possible effectiveness of these transcript variants as diagnostic biomarkers for these particular leukemias.

## 2. Materials and Methods

Subjects: Blood samples were obtained from a total of 32 healthy adult volunteers. These volunteers were selected from King Abdulaziz Medical City (KAMC) and King Abdulaziz bin Saud University for Health Sciences (KSAU-HS). Additionally, leukemic samples were collected from the Hematology departments at KAMC and the Hematopathology department at King Fahad Medical City (KFMC). The leukemic samples were obtained from a total of 45 patients who had been diagnosed with one of the four primary leukemias: AML with 17 samples, ALL with 8 samples, CML with 5 samples, and CLL with 15 samples. The study obtained ethical approval from the Institutional Review Boards (IRB) of both KAMC and KFMC. Furthermore, written informed consent was obtained from all participants involved in the study.

Isolation of PBMC: PBMC were isolated by collecting blood samples with Na-EDTA as an anticoagulant. Following this, 10 mL of the anticoagulated blood was combined with an equal volume of phosphate buffered saline (PBS) and slowly placed on top of 15 mL of Ficol-Hypaque in Leucosep Tubes (50 mL, Greiner Bio-One North America Inc., Monroe, NC, USA). The samples were then centrifuged at 450× *g* using a swing-out rotor for 30 min at 22 °C. Post-centrifugation, PBMC formed a distinct layer above the red blood cell (RBC) pellet. The PBMC band was collected using a pipette and washed several times with PBS. In order to maintain RNA integrity, 50 µL of Qiagen RNALater (Qiagen, Germantown, MD, USA) was added to the PBMC pellet, and the samples were subsequently frozen at −80 °C.

Reverse Transcriptase–Quantitative RT-qPCR Analysis: The Ambion Aqueous kit (Ambion, Austin, TX, USA) was used to isolate the total RNA, and its quality and quantity were evaluated using the Agilent Bioanalyzer 2100 (Agilent, Santa Clara, CA, USA). Following this, 1 μg of total RNA was subjected to reverse transcription using the first-strand cDNA synthesis Kit (MilliporeSigma, Billerica, MA, USA). Real-time RT-qPCR for INSR (IR-A and IR-B) and LMNA/C transcript variants (LMNA, LMNC, LMNAΔ10, LMNAΔ50) was conducted using the 7900HT Fast Real-Time PCR System (Thermo Fisher Scientific Inc., Waltham, MA, USA), as described before [17,30]. All probes were labeled with a fluorescent dye (FAM) for detection and a non-fluorescent quencher (BHQ-1). The sequences for all primer/probe combinations are listed in Table 1. A reaction volume of 20 μL was used for *Taqman* RT-qPCR. The probe reaction assay consisted of 100 mM KCl, 20 mM Tris (pH 9.2), 5 mM MgSO_4_, 0.02% Triton X-100, 0.2 mM dNTP, 200 mM Betaine, 5% DMSO, 1.25 IU Taq Polymerase, 0.2 μM Sense/Anti-sense primers, 0.1 μM Probe, and 2 μL of cDNA. The reaction protocol involved an activation cycle of 50 °C for 2 min, followed by 95 °C for 15 s. Subsequently, 40 cycles of denaturation at 95 °C for 15 s, and annealing/extension (LMNA: 58 °C, LMNC: 60 °C, LMNAΔ50: 60 °C, LMNAΔ10: 66 °C, IR-A: 60 °C, and IR-B: 60 °C) for 2 min were performed. The gene expression levels were standardized by employing Ubiqiotin C and RPL13 as internal control genes, aided by geNORM V3.5 software [40]. 

Statistical Analysis: The statistical analysis was carried out using SigmaStat software version 3.0 (Jandel Scientific, San Rafael, CA, USA). In order to compare normal and leukemic specimens, the Mann–Whitney Rank Sum Test was utilized. Furthermore, the Kruskal–Wallis One Way Analysis of Variance (ANOVA) on Ranks was employed to compare normal specimens with different primary leukemic types. Subsequently, Dunn’s test was conducted for all pairwise comparisons and comparisons against the control group. A significance level of *p* value < 0.05 was used to assess the statistical significance of all analyses. The results are presented as mean ± S.E.M.

## 3. Results

### 3.1. Demographic Data of Subjects

The study included 77 participants, with 32 healthy individuals and 45 subjects diagnosed with one of the four main types of leukemia (AML: 17; ALL: 8; CML: 5; and CLL: 15). The age distribution among the leukemia patients was notably different from that of the non-leukemia group, with the exception of those with ALL (Table 2).

### 3.2. Differential Expression of LMNA/C mRNA Expression in PBMC

**Figure 1:**
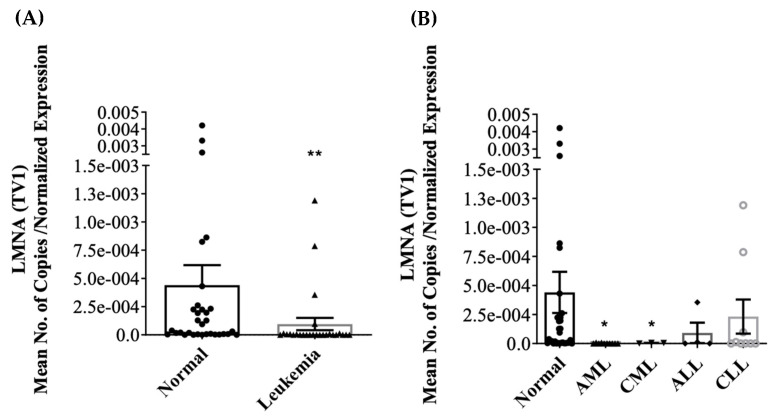
*TaqMan* qRT-PCR measurement of relative mRNA expression levels of LMNA in (**A**) normal versus leukemic subjects and (**B**) following stratification into the four primary leukemias (AML, CML, ALL and CLL). The error bars represent mean ± S.E.M.; ** *p* < 0.001; * *p* < 0.05.

**Figure 2:**
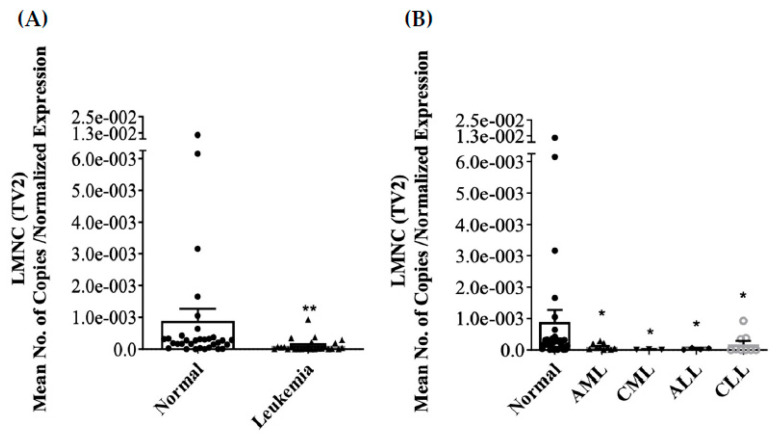
mRNA expression of LMNC in PBMCs of both normal and leukemic subjects. In the case of leukemia, the circulating PBMCs are linked to a decrease in the levels of LMNC mRNA expression (**A**). Furthermore, when stratified into the four primary leukemias (AML, CML, ALL, and CLL), reduced expression of LMNC is observed (**B**). The error bars in the figure represent the mean ± S.E.M., while the symbols ** and * indicate statistical significance with *p*-values < 0.001 and 0.05, respectively.

**Figure 3:**
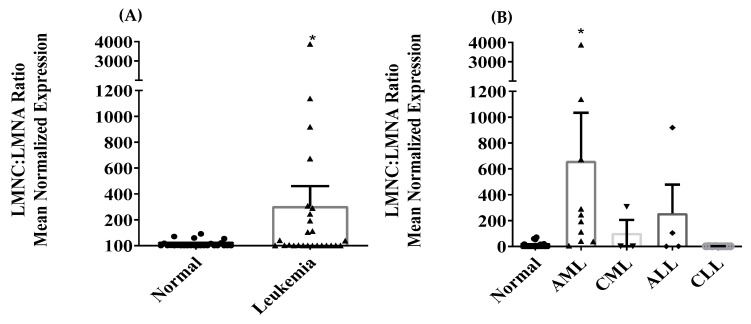
Comparison of the LMNC:LMNA mRNA ratio between normal and leukemic subjects in (**A**), and the LMNC:LMNA mRNA ratio in the same samples after stratification based on the four primary leukemias in (**B**). The error bars in the figure represent the mean ± S.E.M. Furthermore, the asterisk (*) indicates statistical significance with a *p*-value < 0.05.

Differential expression of LMNA/C transcript variants (LMNA (TV1), LMNC (TV2), LMNAΔ10 (TV3), and LMNAΔ50 (TV4 or Progerin)) mRNA was investigated in PBMCs of both normal and leukemic subjects. Only LMNA and LMNC transcript variants were detected in PBMCs. LMNAΔ10 and LMNAΔ50 mRNA were either not detected or had high CT values in a few normal samples. In leukemia, the expression of LMNA mRNA in PBMCs was significantly reduced, as indicated by Figure 1A (*p* < 0.001). The further stratification of leukemic samples into the four primary leukemic types was shown to lead to the significant inhibition of LMNA in AML and CML, as depicted in Figure 1B (*p* < 0.05). Similarly, the expression of LMNC mRNA in PBMC was significantly inhibited in leukemia, as shown in Figure 2A (*p* < 0.001). Moreover, LMNC expression was significantly inhibited in all four primary leukemias (AML, CML, ALL, and CLL), as depicted in Figure 2B (*p* < 0.05). The mean normalized expression levels of LMNA and LMNC were calculated using geNORM V3.5 software. A Mann–Whitney Rank Sum Test analysis comparing normal (n = 32) and leukemia (n = 45) specimens revealed a significant difference between the two groups in terms of the LMNC:LMNA ratio, as shown in Figure 3A (*p* < 0.05). Furthermore, Kruskal–Wallis ANOVA analysis identified significant differences between normal and AML samples when stratified into the four primary leukemia types, as depicted in Figure 3B (*p* < 0.05). Notably, no statistical correlation was found between age and the expression of LMNA or LMNC or the LMNC:LMNA ratio.

### 3.3. Inhibition of INSR (IR-A) mRNA Expression in PBMC

**Figure 4:**
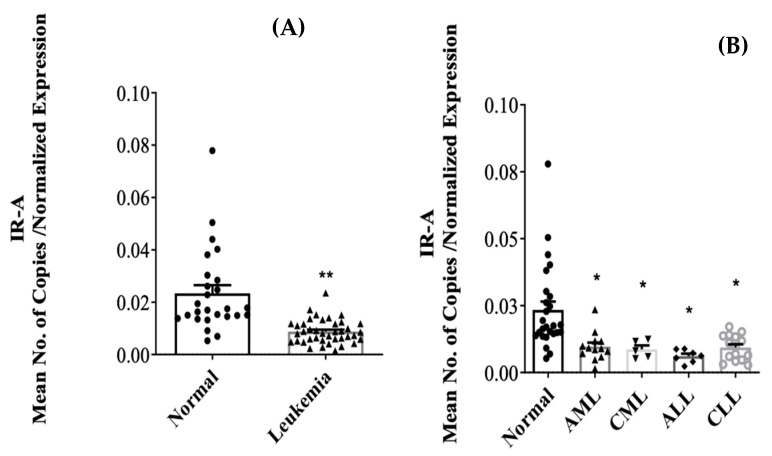
Normalized mRNA expression levels of IR-A in PBMC of both normal and leukemic subjects (**A**). The data are further categorized into normal and the four primary leukemic types (AML, CML, ALL, CLL) (**B**). The error bars in the graph represent the mean ± Standard Error of the Mean (S.E.M.). The significance of the results is indicated by the symbols ** (*p* < 0.001) and * (*p* < 0.05).

The INSR transcript variants, IR-A and IR-B, were assessed in PBMC samples collected from both healthy individuals and individuals diagnosed with leukemia. The IR-A variant (TV2) was present in PBMC samples from both healthy individuals and leukemia patients, while the IR-B variant (TV1) was not detected in PBMC samples from either group, even at a CT value exceeding 45. Interestingly, a notable reduction in IR-A expression was observed in leukemia patients compared to healthy individuals (Figure 4A; *p* < 0.001). Subsequent analysis, categorizing the leukemia patients into the four main types (AML, CML, ALL, and CLL), demonstrated a significant suppression of IR-A expression across all four types (Figure 4B; *p* < 0.05).

## 4. Discussion

The levels of four transcript variants of the LMNA/C gene in humans, namely LMNA, LMNC, LMNAΔ10, and LMNAΔ50 (Progerin), were evaluated using *TaqMan* RT-qPCR assays that were specifically designed to accurately measure their mRNA levels without any cross-reactivity [17]. Among these variants, only LMNA and LMNC were found to be significantly present. Previous studies have reported a decrease in LMNA expression in certain cell subsets characterized by low differentiation and high proliferation rates, including various types of human cancers [41,42]. This reduced expression of LMNA has also been observed in leukemia and lymphoma, often associated with epigenetic silencing through CpG island promoter hypermethylation [43,44,45]. Our findings are consistent with these observations, as we observed notably lower expression levels of LMNA/C variants in PBMCs, particularly in cases of leukemia. This could potentially lead to alterations in cell matrix strength, affecting drug sensitivity and proliferation rates [46].

Despite the sensitivity of our Progerin-specific RT-qPCR assay, we did not detect significant levels of Progerin in PBMCs from normal or leukemic patients. A previous study using the same assay found lower levels of Progerin in breast cancer biopsies compared to normal tissue [17]. Progerin, which is predominantly found in Hutchinson Gilford Progeria Syndrome (HGPS) cells, is typically expressed at very low levels in normal cells, making its detection through RT-qPCR or Western blotting challenging [47,48,49]. The protein expression of Progerin, which is absent at the mRNA level in skin samples from newborns to elderly individuals, increases with age but remains relatively low even in older individuals [50]. These discrepancies may be attributed to the insensitivity of progerin antibodies due to their low abundance, necessitating the use of more sensitive detection methods for Progerin protein. The distinction between Progerin and LaminΔ10 proteins through Western blotting is challenging, as they only differ by 30 amino acids. Similarly, we did not detect LMNAΔ10 expression in PBMC, possibly due to the reduced or absent expression of LMNA/C in PBMCs.

Cytokines play a crucial role in the initiation and progression of various pathological processes, including cancer [51]. These molecules have the ability to either inhibit or stimulate cell growth, regulate cell differentiation, and induce cell chemotaxis. Leukemia originates from immature or developing cells in the bone marrow. In subsets of cells with a low degree of differentiation, the expression of LMNA/C is either reduced or absent. Research has shown that reduced LMNA/C expression in leukemia patients can lead to the abnormal maturation of white blood cells, making them less effective in fighting off infections [52]. This is particularly evident in CLL, where impairments in granulocyte functions contribute to increased susceptibility to infections [53]. Tumor growth can also impede the maturation of natural killer (NK) cells, further compromising the immune system [54]. In myelodysplastic syndrome (MDS), the reduced expression of activating receptors on bone marrow NK cells is associated with the impaired killing of blasts [55]. CLL is also associated with a profound immune defect, leading to increased susceptibility to infections [56]. Furthermore, the overexpression of LMNA/C in macrophages has been found to activate the proinflammatory nuclear factor κB (NF-κB) pathway and increase the expression levels of proinflammatory genes, such as Interleukin-6 (IL-6), tumor necrosis factor α (TNFα), and induced nitric oxide synthase 2 (NOS2) [57]. Conversely, the depletion of LMNA/C in macrophages suppresses the inflammatory responses induced by lipopolysaccharides (LPS) [57]. However, the aforementioned study did not specifically investigate the different transcript variants of *LMNA/C*. Therefore, it is crucial to examine the role of these transcript variants in cytokine production to gain a comprehensive understanding of the mechanism underlying inefficient cytokine production in white blood cells, including those affected by leukemia. These findings underscore the importance of understanding and addressing immune dysfunction in leukemia patients to improve their outcomes. In our study, we observed a downregulation of LMNA and LMNC transcript variants in leukemia, particularly in AML. The ratio of LMNC to LMNA expression may potentially influence cytokine production, as well as cell growth, differentiation, and chemotaxis. Further investigations are warranted to explore the modulatory effect of LMNA/C transcript variants on these cellular functions in the context of leukemia.

The potential of the IR-A:IR-B ratio as a tumor biomarker has been investigated in breast adenocarcinoma [33] and NSCLC [34]. In breast cancer, a decrease in IR-B expression was identified as the primary factor responsible for altering this ratio, while IR-A expression remained unchanged [34]. However, our *TaqMan* RT-qPCR analysis revealed an increase in IR-A expression in breast cancer [30], which is consistent with the elevated levels of IR-A observed in NSCLC [34]. Interestingly, while radioactive insulin binding sites were found on most ALL, AML, CML, and AMoL cells, they were not detected in CLL [26]. This contradicts our findings of IR-A expression in CLL. Our results align with a previous study that demonstrated the absence of IR-B in lymphocytes [28]. Notably, IR-B was absent in PBMC, which consists of monocytes, T and B cells, and other lymphoid-origin cells. Therefore, based on our findings, the IR-A:IR-B mRNA ratio cannot be considered a reliable leukemia tumor marker.

Although neoplastic tissues typically express IR-A [24,26], this study found suppressed IR-A transcript variant expression in AML, ALL, CML, and CLL. The INSR pathway directly impacts cancer development in solid tumors, with insulin and IGF-II activation being prevalent in cancer cells, particularly in dedifferentiated/stem-like cells [26]. INSR and IGF-IR play distinct roles in regulating genes associated with proliferation, apoptosis, differentiation, and cell adhesion [25], and dysregulated IR-A expression plays a crucial role. Historically, IGF-IR has been the primary focus of cancer therapy, overshadowing INSR until recently. INSR facilitates farnesyltransferase activation, which is essential for p21Ras activation via growth factors [58]. LMNA initially exists as a 74-kDa precursor, preLMNA, featuring a C-terminal CaaX motif. Subsequent post-translational modifications such as farnesylation, aaX cleavage, and carboxylmethylation occur, followed by endoproteolytic cleavage by Zmpste24 [59]. Our findings, in conjunction with these discoveries, suggest a potential association between INSR and LMNA/C transcript variant processing. Inhibiting IR-A may impede LMNA and LMNC transcript variant expression, at least in PBMC, possibly by inhibiting farnesyltransferase activation. Further research is necessary to elucidate this connection and investigate the potential of insulin potentiation therapy (IPT) in leukemia treatment. IPT, which involves the use of insulin alongside low-dose chemotherapy, has gained global attention but requires additional investigation to determine its efficacy in treating leukemia.

Numerous investigations have provided evidence of a significant association between higher body weight and the occurrence of leukemia. Prior studies have emphasized an increased risk of leukemia among individuals who are overweight or obese, with the latter group also showing a higher susceptibility to specific subtypes of leukemia [60,61]. Furthermore, another study revealed that excessive weight at the time of diagnosis had a negative impact on the prognosis of pediatric acute lymphoblastic leukemia [62]. In contrast to these findings, our study did not find any substantial disparity in weight between control subjects and individuals diagnosed with leukemia.

The blood contains PBMCs, which are a diverse mixture of monocytes, dendritic cells, lymphocytes (T and B cells), and other lymphoid cells. In myelogenous leukemia, the abnormal cells primarily consist of granulocytes or monocytes, whereas lymphocytic leukemia is mainly composed of lymphocytes. However, PBMCs may not be the most suitable sample for studying AML and CML due to the absence of granulocytes. Moreover, the utilization of heterogeneous PBMCs in this particular study is a significant limitation, and it would be more advantageous to employ purified white blood cells for such investigations. 

To summarize, the presence of LMNA and LMNC mRNA has been observed exclusively in PBMCs. However, the expression of these transcript variants is suppressed in PBMCs of individuals with leukemia. Notably, the ratio of LMNC to LMNA mRNA is notably elevated in AML, suggesting its potential as a diagnostic marker for this type of leukemia. On the other hand, IR-B is not detected in PBMCs, indicating that the ratio of IR-A to IR-B mRNA cannot serve as a diagnostic marker for leukemia. Furthermore, the expression of IR-A is inhibited in various types of leukemia, including AML, CML, ALL, and CLL.

## 5. Conclusions

In conclusion, our research offers significant findings regarding the expression profiles of LMNA/C and INSR transcript variants in PBMCs among individuals with leukemia. We have identified distinct patterns of expression for these variants, with particular attention to the LMNC:LMNA ratio, which holds the potential for diagnosing AML. Furthermore, our results underscore the importance of studying the role of IR-A in the development of leukemia. Gaining a comprehensive understanding of IR-A’s contribution to disease progression is essential for unraveling its mechanisms and exploring potential therapeutic approaches.

## Figures and Tables

**Table 1:** Primer sequences for all primers used in qRT-PCR.

Primer.	Sense (5′→3′)	Anti Sense (5′→3′)	Probe	Accession Number
LMNA	TGACTGTGGTTGAGGACGAC	GACACTGGAGGCAGAAGAGC	CGCTGAGTACAACCT	NM_170707.3
LMNC	GTGGAAGGCACAGAACACCT	GCGGCGGCTACCACTCAC	AGATGACCTGCTCCATCACC	NM_005572.3
LMNAΔ10	AACTCCACTGGGGAAGGCTCC	GCTCCTGAGCCGCTGGCAGA	AGTACAACCTGCGCTCGCGC	NM_170708.3
LMNAΔ50	GCGTCAGGAGCCCTGAGC	GACGCAGGAAGCCTCCAC	AGCATCATGTAATCTGGGACCT	NM_001282626.1
INSR (IR-A)	TATCCGGAACAACCTCACTA	GGAAGAGCAGCAAGTAATCA	CTCTGTCATCGAAGGACACTTG	NM_001079817
INSR (IR-B)	AGGAGTCCTCGTTTAGGAAG	AGGAAGTGTTGGGGAAAG	AGAAAAACCTCTTCAGGCACTG	NM_000208
Ubiquitin C	ACTACAACATCCAGAAAGAGTCCA	CCAGTCAGGGTCTTCACGAAG	CCCACCTCTGAGACGGAGCACCAG	NM_021009.6
RPL13	AACAAGTTGAAGTACCTGGCTTTC	TGGTTTTGTGGGGCAGCATA	CGCAAGCGGATGAACACCAACCCT	NM_000977.3

**Table 2:** Demographic data of the subjects that participated in the study. The results of the study are presented in the form of mean ± S.E.M. Furthermore, statistical significance was determined by comparing the results to the control group (* *p* < 0.05) and the ALL group (^ε^ *p* < 0.05).

	Gender	Age (years)	BMI kg/m^2^
Control	14M, 18F	31.9 ± 2.18	32.1 ± 2.0
AML	9M, 8F	48.2 ± 5.18 *	26.4 ± 1.28
CML	4M, 1F	59.4 ± 6.75 *^ɛ^	30.4 ± 1.78
ALL	5M, 3F	34.4 ± 5.51	25.9 ± 2.38
CLL	7M, 8F	66.5 ± 3.42 *^ɛ^	28.1 ± 1.98

## Data Availability

The data supporting the findings of this study are available from the corresponding author upon reasonable request.
